# Systematic analysis of insertions and deletions specific to nematode proteins and their proposed functional and evolutionary relevance

**DOI:** 10.1186/1471-2148-9-23

**Published:** 2009-01-28

**Authors:** Zhengyuan Wang, John Martin, Sahar Abubucker, Yong Yin, Robin B Gasser, Makedonka Mitreva

**Affiliations:** 1 The Genome Center, Department of Genetics, Washington University School of Medicine, St Louis, MO 63110, USA; 2 Department of Veterinary Science, The University of Melbourne, Werribee, VIC 3030, Australia

## Abstract

**Background:**

Amino acid insertions and deletions in proteins are considered relatively rare events, and their associations with the evolution and adaptation of organisms are not yet understood. In this study, we undertook a systematic analysis of over 214,000 polypeptides from 32 nematode species and identified insertions and deletions unique to nematode proteins in more than 1000 families and provided indirect evidence that these alterations are linked to the evolution and adaptation of nematodes.

**Results:**

Amino acid alterations in sequences of nematodes were identified by comparison with homologous sequences from a wide range of eukaryotic (metzoan) organisms. This comparison revealed that the proteins inferred from transcriptomic datasets for nematodes contained more deletions than insertions, and that the deletions tended to be larger in length than insertions, indicating a decreased size of the transcriptome of nematodes compared with other organisms. The present findings showed that this reduction is more pronounced in parasitic nematodes compared with the free-living nematodes of the genus *Caenorhabditis*. Consistent with a requirement for conservation in proteins involved in the processing of genetic information, fewer insertions and deletions were detected in such proteins. On the other hand, more insertions and deletions were recorded for proteins inferred to be involved in the endocrine and immune systems, suggesting a link with adaptation. Similarly, proteins involved in multiple cellular pathways tended to display more deletions and insertions than those involved in a single pathway. The number of insertions and deletions shared by a range of plant parasitic nematodes were higher for proteins involved in lipid metabolism and electron transport compared with other nematodes, suggesting an association between metabolic adaptation and parasitism in plant hosts. We also identified three sizable deletions from proteins found to be specific to and shared by parasitic nematodes, which, given their uniqueness, might serve as target candidates for drug design.

**Conclusion:**

This study illustrates the significance of using comparative genomics approaches to identify molecular elements unique to parasitic nematodes, which have adapted to a particular host organism and mode of existence during evolution. While the focus of this study was on nematodes, the approach has applicability to a wide range of other groups of organisms.

## Background

Novel molecular signatures specific to particular taxonomic or organismal groups have possible applications for advancing of our understanding of the species within them. In addition, emerging strategies for protein engineering and drug design exploit molecular evolutionary information of proteins [1-6]. Approaches such as targeting evolutionary conserved residues and domains in proteins to modify the interaction of enzymes with other compounds have been reported [2,7-9]. An example is the development of the drug Raltegravir, which targets the conserved reaction core domain of integrase of the human immunodeficiency virus (HIV) [10,11] and provides a treatment for patients with resistance to conventional classes of drugs against human immunodeficiency virus (HIV) infections. However, compared to the focus on conserved proteins, there has been very limited attention to exploring variation in protein sequences and structures linked to insertion/deletion events in homologous proteins among different groups of organisms. Depending on their location and size, insertions and deletions can have a significant effect on the structure and function of proteins, resulting in significant diversity and reflecting the adaptation of an organism to a specific mode of existence or environment [12,13]. Therefore, insertions and/or deletions unique to particular groups of organisms could advance our understanding of such groups at a molecular level and provide useful genetic, biochemical or physiological markers and targets for drug design.

Insertions and deletions (indels) in coding sequences have been reported to be the result of one or more mutation processes, including DNA mispairing [14], crossover [15], transposition [16], and/or others [17]. However, the precise processes and mechanisms remain to be determined. Despite their effects on sequence diversity, indels are considered to be relatively rare events compared to point mutations [18,19]. Indels are also less likely than substitutions to be selectively neutral and are proposed to be under constant selective pressure and are frequently deleterious [20]. Many proteins are under substantial functional and structural constraints, thus limiting substitutions at the amino acid level [21]. Indels accumulated during evolution, not deleterious to a species or a group of species, can change protein structures and function [22,23] leading to adaptations to new environments [24].

The paucity of information on indels, particularly those conserved across different phyla, has limited our understanding of protein function and evolution in relation to the adaptation of organisms to particular environments or hosts. Interestingly, preliminary study of proteins inferred from genomic data sets for parasitic nematodes suggested the presence of nematode-specific proteins and indels compared with other Metazoa (Mitreva et al., unpublished), which emphasizes the need to undertake extensive investigation for different evolutionary groups. A systematic, comparative bioinformatics analysis of sequence and structural differences in proteins could thus provide unique insights into evolutionary and functional aspects of proteins in parasitic organisms, particularly in relation to host adaptation and ecology. From an applied perspective, such analyses could also identify indel events that might be further explored for the rational design of drugs to such unique targets. This latter statement is particularly pertinent to parasitic nematodes, because of widespread problems with drug resistance [25,26] and the need to find new intervention strategies, including drugs and vaccines [27].

Nematodes represent one of the largest phyla of animals, yet many remain poorly described. Thus far, more than 25,000 have been described [28]. Although there are many uncertainties regarding the evolution of Nematoda, a molecular phylogeny, based on the use of a ribosomal subunit gene, has proposed five main clades (I-V) [29,30]. These clades reflect the diversity in biology and ecology within Nematoda and suggest that the adaptation to parasitism has occurred multiple times during their evolution. In contrast to free-living nematodes, such as members of the genus *Caenorhabditis*, many parasitic nematodes cause diseases in plants and animals and are thus of major socioeconomic importance. For instance, hundreds of millions of people are infected with geohelminths (soil-transmitted worms), such as *Ancylostoma duodenale *and/or *Necator americanus*, *Trichuris trichiura *and *Ascaris *spp. [31], causing serious adverse effects on human health, particularly in children. Similarly, parasitic nematodes of livestock, such as cattle and sheep, also cause substantial economic losses worldwide, with billions of dollars spent annually on the treatment and control of nematodes. In addition to the socioeconomic impact that these parasites have, there is potential for the emergence of resistance against the main classes of nematocidal compounds used to treat the diseases they cause [32]. Therefore, there is a significant need to work towards discovering new compounds to control these parasites.

Genomic technologies can assist in this discovery effort. For example, expressed sequence tag (EST) sequencing projects provide a resource for the prediction of new drug and vaccine targets [e.g. [33-35]]. Recently, the projects conducted at Washington University with its collaborators have succeeded in generating ~300,000 expressed sequence tags (ESTs) from 32 species of nematodes representing four of the five clades of the Nematoda. The amount and depth of this dataset enabled us to carry out a systematic analysis of insertions and deletions in proteins inferred from these transcripts by comparing to proteins from a range of other organisms (including a range of metazoans). The present study conducted a comprehensive comparative analysis to identify indels specific to nematode proteins and their relationships with molecular function and organism adaptation. This initial analysis provided a foundation for "indel-based" drug design in nematodes, while elucidating possible mechanisms of nematode parasitism, adaptation and protein evolution.

## Results

Taking advantage of the abundance of nematode sequences accumulated from several nematode sequencing projects, and the large evolutionary distances between some sequences, the present study characterized insertions and deletions (indels) unique to nematode proteins. Distinct from most previous studies [19,36-38], which combined data on indels, the present analysis considered both insertion and deletion events by comparing nematode proteins with those of their homologues in other eukaryotic (metazoan) organisms. We defined insertions unique to nematodes as gaps common to all other eukaryotic sequences, and nematode-unique deletions as gaps absent from any other sequence. To ensure the reliability in the identification of insertions and deletions, we focused the analyses on the 1286 families with 10 or more homologues available from other metazoa and at least one fungus. These families contain 20,695 polypeptides (12,583 inferred from EST contigs and 8,112 from five different genome sequences) in nematodes. The average length of the peptides inferred from EST contigs and genomes was 171 and 456 amino acids (aa), respectively. For a protein family to be considered in the present analysis, it had to represent at least 3 different species (one species of *Caenorhabditis *and at least 2 parasitic species). Hence, only alignments that represented both *Caenorhabditis *and parasitic nematodes were evaluated, thus eliminating bias due to length differences. We studied the evolution of the insertions and deletions in nematodes *via *a detailed analysis of insertion and deletion events that were shared among different lineages or clades. The relationships of these indels were studied by examining the inferred function of their corresponding proteins. Also, the associations between the presence of indels and different tropic ecology (i.e., food source) as well as the parasitic mode of existence were studied.

### Uneven insertions and deletions

**Figure 1:**
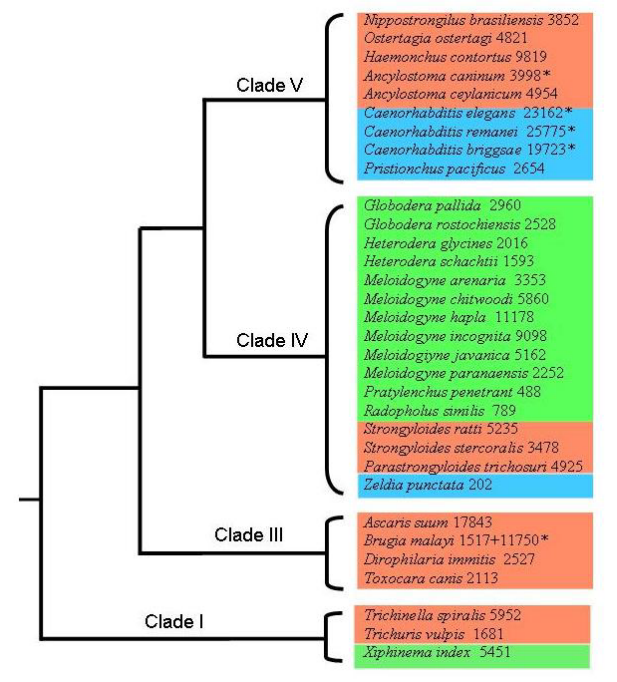
**Nematode species and number of polypeptides per species used in the analysis**. The species are organized based on their SSU rRNA phylogeny [29]. Numbers following the species names are number of EST-derived contigs or full-length proteins (asterix). The color background is based on the trophic ecology: Green, plant parasitic nematodes; Blue, free-living nematodes, Red, animal and/or human parasites.

**Figure 2:**
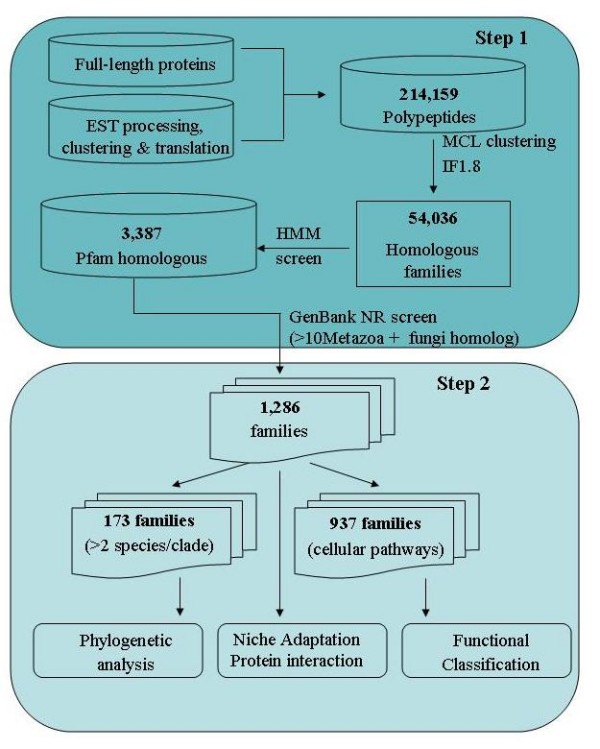
**Identification and analysis of insertions and deletions specific to nematode proteins**. The results obtained from each step of the workflow are shown.

**Figure 3:**
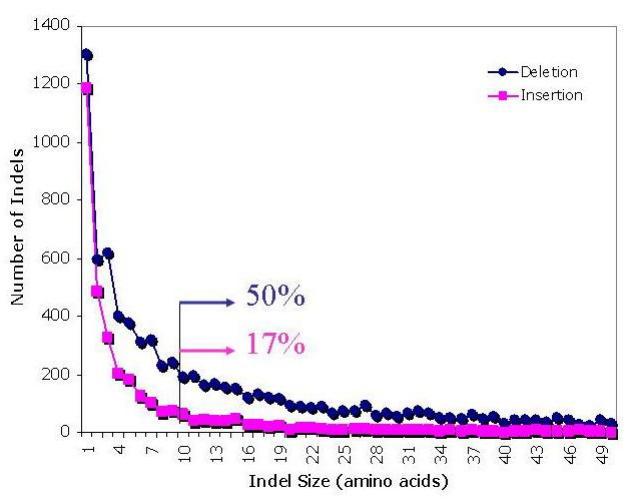
**Frequency distribution of sizes of insertions and deletions**. Fifty percent of deletions are longer than 10 residues, whereas only 17% of insertions are of a comparable length.

EST databases [39] provide a large amount of sequence data for nematodes. A total of 130,357 translations of contig-level EST consensus sequences (comprising 262,497 ESTs) originating from 29 nematode species, were complemented with complete datasets (84,408 proteins) generated in five genome sequencing projects (3 *Caenorhabditis *species, *Brugia malayi*, and *Ancylostoma caninum*). Hence, a total of 214,159 polypeptides/proteins from 32 nematode species were used for the subsequent analysis (Figure 1). The systematic analysis (Figure 2) defined 54,036 homologous families, of which 5,326 were common to at least three species (one *Caenorhabditis *and 2 parasitic nematodes). By focusing on the nematode protein families with homologues in other eukaryotic organisms, we were able to define nematode-specific indels, employing fungal sequences as outgroups. Within these 1,286 families of proteins, the defined number of deletions (10,132) was significantly greater (P < 0.0001, Chi-square) than the number of insertions (3,507). In addition, the deletions (31.3 residues/positions) tended to be longer than the insertions (7.1 residues/position) (Figure 3) (P < 0.0001, Chi-square).

**Figure 4:**
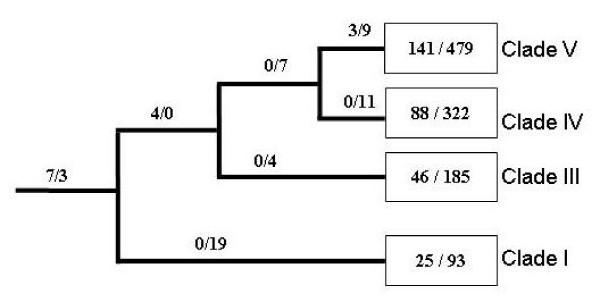
**Distribution of insertions/deletions of 173 protein families that have at least two members per clade**. A total of 2,236 deletions and 636 insertions were identified. ^a^, insertions restricted to this clade; ^b^, deletions restricted to this clade. The species are organized based on their SSU rRNA phylogeny [29].

Subsets of protein families (173 groups) representing at least two species from each clade of the phylum Nematoda (I, III, IV, V) were selected (according to the distribution of member sequences according to species), in order to conduct an analysis of indel events in relation to the proposed molecular phylogeny of Nematoda. For the 173 families, 2,236 deletions and 636 insertions were identified, with a significant bias (P < 0.0001, Chi-square) toward deletions, and, again, the deletions tended to be larger. The indels could be classified into 11 groups based on whether they were common or restricted to clade(s) or lineages (see Methods). The distribution of indels of the 173 families (Figure 4) showed limited numbers of indels at the internal nodes (in terms of common to members from the same clade or different clades), suggesting that the majority of the indels identified evolved after the separation of the clades. The largest number of indels belonged to sequences representing Clade V (Figure 4). For each clade, < 3% of indels were common to all members. Under the assumption that indels shared by all members of a lineage occurred at its last common ancestor (LCA), the present data indicated that shared insertions tended to be more basal than shared deletions (Figure 4). Seven of 14 (50%) of the shared insertions were generated at the LCA, in contrast to < 6% (3 of 53) for deletions (P < 0.0001; Fisher Exact test). More insertions than deletions occurred at the LCA of all nematodes and the LCAs of Clades III, IV and V, whereas more deletions occurred across all other lineages, which is consistent with the tendency of nematode proteins to have more deletions than insertions.

### Evidence of a link between indels and the adaptation from free-living Caenorhabditis to a parasitic mode of existence

**Figure 5:**
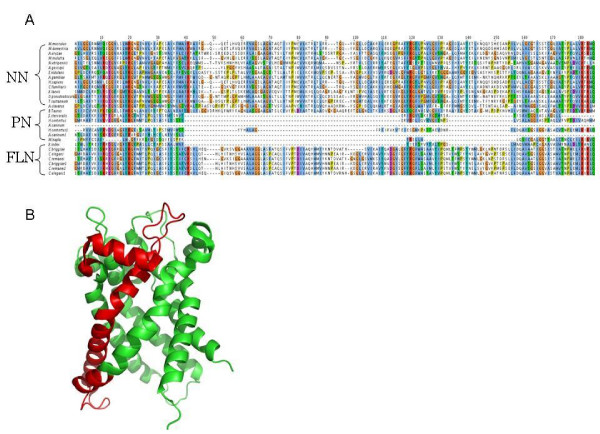
**Characteristics of protein family NF_0309_0623**. A. Multiple alignment of the members of this protein family (the alignment has been trimmed for a better view). This family has proteins that originate from species spanning 3 clades. It encodes mitochondrial carrier protein that belongs to MC family, involved in Environmental Information Processing (based on Panther; [92]). Accession numbers of sequences used are: XP_746931.1, CAD60708.1, XP_427233.2, NP_729803.1, XP_394090.1, CAG07711.1, XP_715902.1, XP_217310.3, XP_001387442, XP_001492793, XP_001649449, NP_001039791, XP_001247964, NP_001008081.1, XP_001163052, NP_998284.1, EDL38228.1, XP001376701, BAE61781.1, XP_001084129, XP_001604399, XP_001374602, NP_984088.1, AAB17185.1, XP_308217.3, EAW69088.1, XP_854738.1, NP_001085887, XP_001361631, XP_971944.1, XP_001273074. B. Structure model 2C3E from *Bos taurus *mitochondrial ADP/ATP carrier was used to show the nematode-specific deletion (marked in red).

We investigated indels based on their trophic ecology, i.e. FLN (Free-living Nematodes representing predominantly the *Caenorhabditis *lineage), PPN (Plant-parasitic Nematodes), and APN (Animal-parasitic Nematodes). These three groups of nematodes had a limited number of common indels (Table 1). The free-living Caenorhabditis nematodes had in common ~10% of deletions and ~30% of their insertions. Interestingly, free-living nematodes had more insertions compared with parasitic nematodes (P < 0.0001, Chi-square) (Table 1). The ratio of insertions to deletions for free-living nematodes was 0.44, whereas for nematodes parasitic in animals and plants, the ratios were lower (0.26 and 0.25, respectively). Also, *Caenorhabditis *spp. shared more insertions (213) than deletions (159). There were only three deletion events (> 10 residues) and one insertion event (1 residue) common to all parasitic nematodes. The proteins associated with the shared insertion were inferred to be involved in fatty acid synthesis (3-oxoacyl- [acyl-carrier protein] reductase). The three deletion events were linked to proteins associated with genetic information processing (26S proteasome regulatory subunit N, and small subunit ribosomal protein S14), and environmental information processing (mitochondrial carrier protein). Because these deletion events were shared only by parasitic nematodes representing different clades, they are likely linked to an adaptation to parasitism. To examine whether these shared, unique indels might represent novel molecular features that warrant further exploration (e.g. targets for parasite intervention), we mapped (as an example) the deletion of the protein family homologous to mitochondrial carrier protein to the structure of its bovine homologue [40]. Figure 5 shows that there is a deletion of an entire helix, including its terminal loops, from the nematode proteins. Unfortunately, there is no structural data available for the other protein families.

**Table 1:** Common^a^/restricted^b ^insertions and deletions in proteins inferred for nematodes with different trophic ecologies

	Free-Living nematodes *Caenorhabditis *species	Plant-parasitic nematodes	Animal/human parasitic nematodes	All Parasitic nematodes
Insertions	213/831	26/340	10/561	1/901
Deletions	159/1894	81/1285	59/2267	3/3552

^a^, common indels, conserved among all members of a group and specific to that group of nematodes,

^b^, restricted indels, restricted to a group of nematodes but not conserved among all of its members.

Interested in proteins with common indels that might relate specifically to a parasitic mode of existence, we classified the protein families with indels common to FLN, PPN, or APN, according to the Panther protein classification system. Table 2 lists the top ten processes based on the number of assigned protein families and grouped them according to the trophic ecology of the species (a complete list of the mappings to the Panther protein classification system is available as Additional file 1). While the highest percentage of families were mapped to "nucleotide metabolism", there was no significant difference among the three different types of nematodes. In contrast, there was a significant difference among nematodes in the number of mappings to the electron transport and lipid metabolism (P < 0.05). In both these categories, PPN had an increased number of families compared with FLN and APN. Approximately 22% of the PPN protein families were associated with these two functional classes, compared with < 10% for free-living and animal-parasitic nematodes (statistically significant difference at P < 0.01, Chi-square).

**Table 2:** Functional classification^a ^of protein families^b ^bearing indels conserved across members of nematodes

	Free-Living	Plant-parasitic	Animal-parasitic	P-value
	(227 families)	(82 families)	(53 families)	(Exact test)
Electron transport	1.30%	6.00%	0%	0.04
Signal transduction	9.30%	8.50%	3.80%	0.51
Carbohydrate metabolism	3.50%	8.50%	3.80%	0.21
Lipid, fatty acid, and steroid metabolism	6.60%	15.90%	5.60%	0.04
Proteolysis	7.00%	10.00%	3.80%	0.46
Protein modification	7.50%	8.50%	7.60%	0.96
Nucleoside, nucleotide, and nucleic acid metabolism	28.20%	28.00%	34.00%	0.58
Intercellular protein traffic	8.80%	6%	3.80%	0.48
Cell cycle	11%	8.50%	17.00%	0.31
Transport	4.40%	4.80%	3.80%	1.00

^a^, Functional classification is based on the Panter protein classification system [92];

^b^, Only proteins families with indels common to a nematode group are included.

### Mapping indel events identified in protein families to cellular pathways

The frequencies of indel events in particular protein families involved in cellular pathways were inferred, in order to explore possible links to biological function and the adaptation of nematodes to their environment and/or hosts. The KEGG pathway database enables associations to metabolic and regulatory pathways, and was therefore used for this analysis. According to KEGG analysis, four main types of pathways were identified: genetic information processing, cellular processes, metabolism, and environmental information processing. The lowest frequencies of insertions and deletions were linked to proteins involved in genetic information processing, whereas the highest frequencies were associated with cellular processes (Table 3), with a difference of ~50% between them. The differences among deletions and insertions were statistically significant (P < 0.05 and P < 0.1, respectively; T-test). A complete list of all KEGG mappings is available as Additional file 2. Examining sub-pathways, transcription was the least represented (4.28 deletions and 2.15 insertions), whereas endocrine signaling and immune system had the highest representation (11.90 deletions and 4.08 insertions; and 11.70 deletions and 3.96 insertions, respectively) (Additional file 3). The differences in frequency and size of indels within proteins involved in single pathways *versus *proteins involved in multiple pathways were also investigated. This analysis included > 300 families homologous to multi-pathway proteins and > 600 families homologous to other proteins, and revealed that the average frequencies of both insertions and deletions of the multi-pathway families were ~30% higher than those of others (insertions: 3.01 *versus *2.42; deletions: 8.79 *versus *6.29) (Additional file 4) and the differences were highly significant (P < 0.0001; T-test).

**Table 3:** Rates of insertions and deletions (average number of nematode specific insertions and deletions per family) of proteins categorized according to biological pathway

	Genetic Information Processing	Cellular Processes	Metabolism	Environmental Information Processing
Insertions	2.54	3.28	2.61	3.07
Deletions	6.17	9.13	7.68	8.38

## Discussion

The present, systematic analysis of indels in proteins of nematode origin has provided useful information to make inferences about protein evolution and function and could have important implications for the identification of novel genetic, biochemical or physiological markers and targets for drug design.

It is known that indels are distinct from point substitutions, and their evolution is affected by distinct influences [41]. The present results support the proposal that insertions and deletions relate to distinct evolutionary processes. The number of deletions is significantly higher than that of insertions, and the sizes of deletions are larger than those of insertions. A previous study also found more deletions in coding sequences in mammals [42]. It appears that mutational bias is one cause of these increased deletions. Analyses of non-coding genomic sequences have revealed a mutational process biased toward deletions [43,44], and the present results indicate that similar mutational mechanisms might apply to the protein-coding sequences. However, the observed larger size of deletions cannot be explained by mutational bias, since deletion and insertion mutations have similar sizes [45]. So, the role that natural selection and adaptation have played here is unclear. From the structural perspective, proteins are more flexible with insertions than deletions [46], although single-residue deletions can be tolerated [47]. Therefore, deletions, particularly larger ones, are not expected to be maintained, which contrasts current observations. It is likely that function-related selection has played a role. Such selection has been identified in a primate sperm ion channel protein [48]. Both the increased number of deletions at the terminal nodes (Figure 4) and the increased common deletions (Table 1) among parasitic nematodes further suggest that the function-related selection is associated with recent species adaptation. Nonetheless, the observed, larger number of deletions and their larger sizes (compared with insertions) indicate a size decrease in nematode transcriptomes during evolution. This size reduction in nematode transcriptomes seems to be consistent with a tendency for their genomes to be smaller than some other metazoa, such flatworms [49] and birds [50].

The biased distributions of indels on different functional pathways (Table 3) further demonstrate the selective forces on them, and suggest the association between increased indels and functional adaptation. Proteins involved in genetic information processing are believed to have stringent selective constraints, and are under strong "purifying selection" [51,52]. Accordingly, these proteins have the least number of insertions and deletions per protein (Additional file 4). Depending on the function, some proteins are under positive selections and accumulate more insertions and deletions [53]. The present study showed that nematode proteins involved in cellular processes (Table 3, Additional file 2) including endocrine signaling pathways and immune system (such as Toll-like receptors and antigen processing) had 50% more deletions and insertion than those involved in genetic information processing. This information agrees with the findings of previous studies [53,54]. Rapidly evolving genes are also considered to be frequently associated with the immune and endocrine systems in other organisms [54,55]. These systems are considered to be key to a specific molecular interaction with the environment where a rapid adaptation to a food source [56] or host may be required. Overall, the present results suggest that proteins bearing sizable nematode-specific indels are functionally grouped. Furthermore, adaptation can lead to an increase of protein sequence changes, including substitution, insertion, and deletion [55,57]. The high rates of insertion and deletion events in proteins involved in multiple pathways might also be viewed as an evidence of functional adaptation, as suggested by recent research [58]. Although a relaxed selective pressure can also lead to such high rates, it is unlikely here. The selective pressure on these proteins is assumed to be greater because they are involved in multiple pathways and potentially interact with more proteins. Their substitutional rates tend to be lower [59]. Thus, increased indels in proteins involved in multiple pathways is suggested to be due to their positive role in an adaptation of nematodes to their host and environment.

More direct evidence for roles of nematode-specific indels in adaptation comes from a comparison of their distributions in different groups of nematodes. The number of indels common to plant parasitic nematodes, compared with other nematodes, is higher for proteins involved in electron transport and lipid/fatty acid/steroid metabolism (Table 2). The higher number could be related to the adaptation of PPN to their specific lifestyle, which includes different stages capable of surviving aerobic and anaerobic environments. Adaptation of these energy metabolism related functional classes is important for parasites. Direct biochemical evidences of adaptation with these two classes have been observed [60-62]. These detected common indels can be one of the reasons for those observed biochemical changes, and suggest that PPN are likely to use indels as a strategy for their adaptation as well as lateral gene transfer [63]. Nonetheless, given the nature and potential bias of our data, the upcoming parasitic nematode genome data [64] will enable us to perform more comprehensive studies which will lead to more firm conclusions.

There is a very limited number of indels occurring on internal nodes (Figure 4), although these internal nodes do not represent short evolutionary times. For example, the internal branch leading to the split of Clade V, IV and III stands for more than 100 million years [65] (the branch leading to Clade III stands for about 350 million years [29,30]). If all indels were retained and their rates are constant, the numbers of indels of these internal branches are expected to be in the same order of magnitude as the terminal branches. The extremely low observed numbers of insertions and deletions on internal nodes could reflect that nematodes have higher indel rates, and thus the number of newly generated indels is higher that the older indels. Thus, sequence comparisons can only detect fewer shared indels. However, it is also possible that an indel burst occurred in the evolution of the Nematoda during a recent adaptation to their life niches.

In addition to insights into fundamental aspects of indel events in proteins, including their evolution and roles in adaptation, this study might assist in the design of intervention strategies against nematodes. Indels common to all parasitic nematodes could provide drug targets for broad control. On one hand, if they are restricted to nematodes, specific targeting will not affect the regular functionality of the homologous proteins in the hosts. Importantly, these indels might also be crucial for the survival of parasites (or they will not be shared by all the parasites since parasitic adaptations are proposed to have occurred several times independently [29]). Using indels to design effective drugs has been successfully explored by Nandan et al., [66] who designed a compound that targeted a 12 residue deletion in the EF-1α protein of the protozoan parasite *Leishmania donovani*. The compound attacks the parasite by blocking this protein without affecting the human homologue. It is likely that the indel used for drug design should be located on the surface of a protein or should relate to a unique structural component of a protein. Sizable deletions uniquely shared by all parasites could possess similar characteristics and become a target for these approaches as well. One such example is the deletion of an entire helix on a functionally important mitochondrial carrier protein (Figure 5). Hence, the identified conserved nematode-specific molecular signatures have possible applications for advancing our understanding of the nematodes.

## Conclusion

Genomics studies of parasites in the phylum Nematoda have been mainly restricted to EST-derived partial proteomes [35,39]. The analysis varied from comparison between two species, to a pan-phylum analysis. Numerous laboratories around the world have contributed to > 520,000 ESTs from more than 40 species. Nematologists currently also have available genome sequences from four nematode species including two parasites. The first annotated genome of a parasitic nematode, *Brugia malayi*, contained over 11,000 genes [67] and the plant parasite *Meloidogyne incognita *over 19,000 genes [68]; both studies have identified potential new anti-parasitic drug targets. However, as the cost for the next-generation sequencing technologies decreases substantially, the sequencing of complete genomes of many eukaryotic species, including parasitic nematodes selected mainly due to their importance to health, evolution and ecology, can be foreseen in the near future. In the next five years, collaborative projects at the Genome Center at Washington University and the Wellcome Trust Sanger Institute will increase the available parasitic nematode sequences by another order of magnitude, adding a total of 25 draft genomes supplemented by millions of cDNA reads *via *pyrosequencing. However, we anticipate that complete annotated genomes for parasitic nematodes are still 2–4 years away. Until then, the transcriptomic data will remain the main source of information for the investigation of nematodes at the molecular level. These EST-derived partial proteomes represent mainly abundantly transcribed sequences and therefore lack or have reduced representation of some gene classes and are thus inappropriate for certain type of analyses, such as "gene loss" which is due to lack of evidence rather than true loss.

However, several pan-phylum analyses have been facilitated by the wealth of transcriptomic data from over 30 nematode species spanning the phylum Nematoda. The first pan-phylum analysis [35], was one of the most complete analyses of the genetic diversity of a single phylum yet attempted, and was full of surprises. This report showed that the *C. elegans *genome contains only a relatively small fraction of the genetic diversity of nematodes. Despite the availability of the genomes of two *Caenorhabditis *species and numerous ESTs, the nematode gene-space appears far from thoroughly sampled because the addition of each new species to the analysis yielded a linear increase in new gene discovery. The same dataset of 265,494 ESTs clustered into 93,645 genes and comprised of 25,871,325 codons were used for a comprehensive codon usage analysis [69]. This latter study demonstrated that codon usage similarity in Nematoda usually persists over the breadth of a genus but then rapidly diminishes even within each clade. This analysis together with an analysis by Cutter et al. [70] based on the same dataset, established the major evolutionary forces responsible for determining observed codon usage bias in nematodes including directional mutation pressure, translational efficiency, and effective population size. Finally, recently Wasmuth et al. [71] have analyzed nematode protein families and distinct domains and found preponderance of genetic novelty in the phylum, i.e. an ongoing "invention of novelty".

These pan-phylum analyses suggest that Nematode proteins and their transcriptomes have experienced drastic changes, and specific changes can be related to functional diversification, speciation and species adaptation [72-76]. Among the nematode-derived proteins, there are two intriguing groups, (i) nematode-specific proteins, which are inferred to be of crucial importance for understanding nematode evolution and parasitism [77-79]; (ii) proteins that have sufficiently diverged in the host as to be functionally absent or altered. The current pan-phylum study has focused on systematic analysis of the latter group and identified a biased distribution in molecular features (insertions and deletions) in the nematode proteins, which assist in elucidating the mechanism of genome size decrease in nematodes. By examining the insertions and deletions common to different nematodes and the functions of the relevant proteins, the present study infers an important role of indels in nematode adaptation. The detected indels shared by different groups of nematodes warrant further investigation as potential targets for the development of compounds against parasitic nematodes.

## Methods

### Data sets and classification of protein families

Sequence data from ESTs or genomic sequencing projects are available for more than 30 species of nematodes (e.g. [35,39,69,80] representing clades I, III, IV and V (cf. [29]) [81]. In the present study a total of 130,357 contig-level EST consensus sequences (representing 262,497 ESTs from organisms other than *Caenorhabditis*) originating from 29 nematode species http://www.nematode.net, were complemented with complete datasets (84,408 proteins) generated in five genome sequencing projects [3 *Caenorhabditis *species http://www.wormbase.org, *Brugia malayi *[67], and *Ancylostoma caninum *[82]. Hence, a total of 214,159 polypeptides/proteins from 32 nematode species were used for the subsequent analysis (Figure 1). Using the Markov Cluster Algorithm (MCL) [83], protein families were defined (Figure 2). To ensure generality of protein families, we required every family to contain member sequences from one species of *Canenorhabditis *and two parasitic nematodes. Representative proteins and protein families for insertion/deletion analysis were selected based on the protein profile model of the family, established using the program hmmpfam from the HMMER package [84]. The member sequence with the highest fitting-score to the family profile model was selected as the representative of the family. Representative peptide sequences were initially screened against the KEGG [85] and Pfam [86] databases to identify significant homology to known proteins. Those without significant homology were eliminated. Representative sequences of the remaining families were then compared against full-length proteins from organisms other than nematodes that were publicly available in the NCBI database (http://www.ncbi.nlm.nih.gov; threshold of 1.0e-3). Homologous sequences originated from metazoans were downloaded into a separate database [henceforth referred to as "reference" (r-) sequences]. Groups of proteins comprising less than 10 reference sequences were excluded, as were those without fungal references. The final dataset contains 1286 protein families with sequences from at least 3 nematode species (one Caenorhabditis and two parasitic species) and not less than 10 other metazoan homologues (including fungus).

### Multiple-alignments and identification of insertions and deletions restricted to nematodes

Alignments of family member sequences with reference sequences were conducted in a step-wise fashion. First, the reference sequences within each protein family were aligned using MUSCLE [87]; second, the nematode sequences of each family were aligned to their reference sequences using CLUSTALW [88], employing the profile alignment methodology [89]. The sequences were aligned using this approach to avoid ambiguity and minimize alignment gaps and maximize similarity/homology. Following the alignment of sequences, family member sequences were edited further by collapsing contigs from the same cluster (i.e. gene) and merging sequences from the same species that shared a significant identity (more than 90 bases or 50% of one sequence). Insertions and deletions unique to nematodes were identified based on the adjusted alignments. The gaps absent from reference sequences were recorded as being nematode-specific gaps. The positions corresponding to the gaps shared by all reference sequences but not any of the member sequences (of a family) were recorded as insertions unique to nematodes. If gaps in different sequences overlapped by more than one third of their total length or more than half of the length of any individual gap, they were treated as "shared gaps". The length of the gap was represented by the average length of their individual member gaps. Insertions (n = 3,507) and deletions (n = 10,132) identified to be unique to nematode protein sequences were subjected to functional and phylogenetic analyses. All analyses were automated using Perl scripts. These scripts are available from the authors upon request.

### Evolution of insertions and deletions

For the evolutionary studies, only protein families with at least two members representing each of the clades I, III, IV, and V were investigated. The indels of these families were analyzed based on the proposed molecular phylogeny of the phylum Nematoda [30,90]. Because these families have multiple sequences derived from each of the four clades, we could identify whether an indel was restricted to members from one clade or was common to multiple clades. Furthermore, we were able to distinguish whether an indel was shared by all members of a specific clade or group of clades (common indels), or whether it was not shared by all members of a designated group but restricted to a lineage (restricted indels). In this way, the indels were classified into 11 groups (common or restricted to members from each of the four clades, restricted to clade III, IV and V, restricted to Clade IV and V, and common to all four clades). The numbers of insertions and deletions in each of these groups were calculated. Given the phylogenetic relationships of these clades, it was possible to map the indels of these groups to the proposed phylogeny. For example, those common and restricted to Clade V could be located to the last common ancestor (LCA) of Clade V, while those common to Clade V but not shared by all the member sequences derived from this clade (restricted) might be localized to a descendant in Clade V.

### Insertions and deletions specific to parasitic and/or free-living nematodes

Parasitic nematodes can be grouped into those infecting plants (PPN, plant parasitic nematodes) and others infecting animals, including humans (APN, animal parasitic nematodes). Indels were examined to determine whether they were associated with either or both of these groups. The five free-living species (FLN, free-living nematodes) were used as a control (Figure 1). Furthermore, we made the distinction between indels shared between all the members of a group (common indels) or only by some members (restricted indels). Hence, the indels were categorized into eight classes: indels common to PPN, APN, or FLN, indels restricted to PPN, APN, or FLN, indels common to all parasites, and indels common to all nematodes (including free-living species). The representative sequences of the protein families containing these indels were also compared against data in the Uniprot database [91] to identify homologues for functional classification using the Panther database [92]. The Uniprot homologues possessing these indels were used to explore the function of these families by referring to the functional annotation of proteins by employing Panther. The biological processes that the proteins were predicted to be involved in were then compared.

### Linking insertions and deletions to cellular pathways

Proteins and their cellular pathway annotations were retrieved from the KEGG pathway database http://www.genome.jp/kegg/pathway.html. Protein family members were assigned to cellular pathways based on their homology (cut-off: 35 bits and 50% of identity) to sequences in KEGG (v.44) using WU-BLAST. The "closest" homologue was selected for each family, and insertions and deletions unique to nematodes were then linked to the pathway to which the best homologue could be assigned. Total numbers of insertions and deletions and the mean number of families associated with each pathway were calculated.

Proteins involved in multiple pathways have more interactions with other proteins and tend to be functionally essential [58,59]. Herein, "multi-pathway proteins" were defined as those involved in several cellular pathways, and single-pathway proteins as those involved exclusively in one. Families of "multi-pathway proteins" were identified by examining the annotations of their closest KEGG homologues. If they could be assigned to multiple pathways by KEGG associations, the family was classified as multi-pathway, and if only one pathway could be assigned, the family was classified as a single-pathway protein family. The numbers of nematode-unique insertions and deletions linked to a multi-pathway or single-pathway protein group were calculated and compared.

## Authors' contributions

ZW and MM conceived and designed the experiments. YY, JM, SA and ZW carried out experiments and analyses. ZW, RBG and MM interpreted results and prepared the manuscript. All authors have read and approved the final manuscript.

## Supplementary Material

Additional file 1 **Panther based classification of proteins bearing nematode specific indels**. The data provided represent summary of the functional classification of the proteins bearing nematode specific indels based on the Panther protein classification system. Click here for file

Additional file 2 **KEGG based classification of proteins bearing nematode specific indels**. The data provided represent summary of the pathway associations of the proteins bearing nematode specific indels based on the KEGG pathway information. Click here for file

Additional file 3 **Rates of insertions and deletions of proteins involved in single or multiple pathways**. The data provided are the rates in terms of the number of insertions or deletions within the proteins involved in single or multiple pathways by the numbers of protein families. Click here for file

Additional file 4 **Sizeable indels (> 4 aa) in proteins involved in cellular pathways (classification based on KEGG)**. Summary of the identified indels within proteins involved in cellular pathways. Only indels with ≥ 4 aa are considered. Click here for file
